# Variations in Soil Nitrogen Mineralization Are Associated with Fungal Communities Across Broad-Leaved Forests in Northeast China

**DOI:** 10.3390/plants15142138

**Published:** 2026-07-10

**Authors:** Xu Cao, Lei Guo, Ruihan Xiao, Kexin Tong, Tao Liu, Minghan Lang, Beixing Duan

**Affiliations:** 1 School of Hydraulic and Electric Power, Heilongjiang University, Harbin 150080, China; coxi@s.hlju.edu.cn (X.C.); guolei@s.hlju.edu.cn (L.G.); 2022074@hlju.edu.cn (K.T.); 2002219@hlju.edu.cn (T.L.); 2 International Joint Laboratory of Hydrology and Hydraulic Engineering in Cold Regions of Heilongjiang Province, Harbin 150080, China; 3 Post-Doctoral Mobile Research Station of Ecology, Heilongjiang University, Harbin 150080, China; 4 Liaoning Zhanggutai Desert Ecosystem Research Station, Liaoning Institute of Sandy Land Control and Utilization, Fuxin 123000, China; 18804502009@163.com

**Keywords:** broad-leaved forests, nitrogen mineralization, soil physicochemical properties, microbe community, Northeast China

## Abstract

Soil nitrogen (N) mineralization plays a pivotal role in regulating N availability in forest ecosystems, which could not only be closely related to soil nutrient supply capacity but also profoundly affect the forest carbon sequestration. Broad-leaved forests play a key role in terrestrial carbon storage; however, soil net N mineralization rates (Rmin) vary considerably among different forest types and their underlying driving mechanisms remain poorly understood. In this study, three typical broad-leaved forests in Northeast China, namely *Populus davidiana* Dode forest (PF), *Fraxinus mandshurica* Rupr forest (FF), and *Betula platyphylla* Suk. forest (BF), were selected. The soil Rmin, environmental parameters, physicochemical properties, and microbial community characteristics were determined among the three broad-leaved forests to explore forest type differences in soil Rmin and their associated factors. The results showed that soil inorganic N contents differed significantly among the three forest types, with significantly higher values in FF than in PF and BF (*p* < 0.05). Soil Rmin also differed significantly among forest types, which was highest in FF, followed by PF and BF (2.17, 1.31, and 0.95 mg kg^−1^ day^−1^, respectively) (*p* < 0.05). Soil Rmin was significantly positively correlated with soil water content (SWC), soil temperature (ST), and pH, but there was a negative correlation to soil bulk density (BD) (*p* < 0.05). In addition, microbial biomass carbon, nitrogen, and phosphorus were significantly higher in FF than in PF and BF (*p* < 0.05). Variation in soil Rmin among three broad-leaved forests was significantly associated with the abundances of *Ascomycota*, *Basidiomycota*, and *Mucoromycota*, but not with bacterial community, suggesting a closer association between fungi and soil Rmin. Structural equation modeling (SEM) indicated that forest type was associated with soil microbial community structure and biomass through associations with soil environmental and physicochemical properties, in relation to soil Rmin. In conclusion, this study highlights the links between vegetation type and soil Rmin in broad-leaved forests, which deepens the theoretical understanding of forest soil N-limitation in Northeast China.

## 1. Introduction

As one of the most important components in terrestrial ecosystems, forests play pivotal roles in regulating global nutrient cycles. The nitrogen (N) cycle is the core in the forest nutrient cycles, which could sustain forest ecosystem stability through a series of biochemical processes (i.e., nitrogen fixation, ammonification, nitrification, and denitrification) [1]. However, global forests are generally N-limited, which constraints both vegetation growth and ecosystem functions. Soil N mineralization is a key process in the N cycle, converting organic N into inorganic forms and directly determining soil nutrient availability. The relative lower soil net nitrogen mineralization rate (Rmin) is a key factor leading to plant N-limitation [2,3]. Globally, compared to coniferous forests, N-limitation is less overall in broadleaf forest ecosystems [4]. However, soil Rmin also varies significantly across different broad-leaved forests and such variations remain poorly understood at present. Therefore, it is important to explore the characteristics of soil Rmin and their regulatory mechanisms among different broad-leaved forest systems, which could further deepen our understanding of the forest soil nitrogen cycle.

In recent years, studies on the differences in soil Rmin and its key controls across broadleaf forests have received more and more attention [5,6]. Researchers have suggested that forest type could affect soil Rmin by regulating soil organic carbon (SOC), total nitrogen (TN), pH, C:N ratio, soil temperature (ST), and soil water content (SWC) [7,8,9]. However, due to the differences in climate, vegetation, and soil types, no consensus exists regarding the major regulatory factors controlling soil Rmin across different broadleaf forests. Zhao et al. reported that the differences in soil Rmin among broadleaf forests were closely related to SOC and TN contents [10]. Prior research has highlighted soil pH as a key factor controlling Rmin and suggested that near-neutral soils are more favorable for N mineralization than acidic soils [11,12,13]. Also, the important role of soil C:N ratio in regulating Rmin has been reported [8,14]. Meanwhile, the interaction effects of multiple factors on Rmin should not be ignored. For instance, a study in subtropical broad-leaved forests suggested that ST was the primary driver of soil Rmin at lower ST conditions (<25 °C) [15], while SWC became the dominant factor at higher ST conditions (≥25 °C) [16]. Therefore, elucidating the key factors governing Rmin is essential for understanding the N cycle mechanisms in broadleaf forest soils.

Soil microbes are key regulators of forest soil N cycling, with their community structure, diversity, and richness collectively regulating Rmin [17,18]. Tree species identity is a key driver of soil microbial community composition and diversity [19,20]. Vegetation species could shape microbial community structure and function by affecting soil microenvironment and substrate availability [7,21,22], and ultimately influences Rmin. Most studies have revealed that soil bacteria are the core microbial community driving soil Rmin in different broadleaf forests [23,24]. Specifically, shifts in the abundance of *Proteobacteria* directly correlate with Rmin [25]. In contrast, Ferreira et al. argued that *Actinobacteria* and *Acidobacteria* were key phyla regulating Rmin among different broadleaf forests [26]. Furthermore, soil fungal communities also play vital roles in regulating Rmin [27,28]. Some studies have suggested that *Ascomycota* is a key fungal community in regulating soil Rmin [29,30]. At the same time, microbial biomass also exerts notable effects on Rmin [31,32,33]. Therefore, the microbial regulatory mechanisms of Rmin in different forest types remain poorly understood, and further study is still needed.

Temperate broadleaf forests represent a key component of regional forest ecosystems in Northeast China. As an ecological barrier for the Northeast China Plain and the North China Plain, broadleaf forests contribute substantially to regional climate regulation and ecological security [34,35]. Under global climate change, warming may alter plant growth and nutrient demand in forest ecosystems [36]. However, the widespread N-limitation in forest ecosystems here may inhibit the response of plant growth to warming, and ultimately influence the ecosystem functions [37,38]. Soil N mineralization is a critical process, regulating the production of plant-available N and therefore playing an important role in determining soil N availability and forest nutrient cycling [39,40]. Thus, understanding soil Rmin and its regulatory mechanisms is essential for evaluating soil N supply capacity in the temperate broadleaf forests of Northeast China. In the present study, three typical broadleaf forests in Northeast China, namely *Populus davidiana* Dode forest (PF), *Fraxinus mandschurica* Rupr forest (FF), and *Betula platyphylla* Suk. forest (BF), were selected. The soil Rmin and its relative factors were determined among three broadleaf forests to clarify their N transformation characteristics and associated regulatory mechanisms. We hypothesized that (1) there exist notable differences in soil Rmin and N availability among different forest types, (2) such differences may be tightly linked to variations in soil microbial communities (bacteria and fungi) and are jointly regulated by environmental factors and soil properties.

## 2. Results

### 2.1. Soil Inorganic N

**Figure 1:**
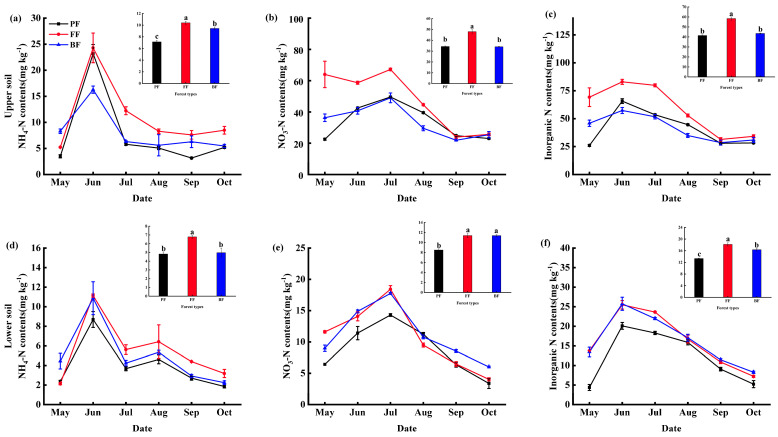
Seasonality dynamics of soil NH_4_^+^-N contents (**a**,**d**), NO_3_^−^-N content (**b**,**e**), TIN contents (**c**,**f**) among different types of forest at soil depths of 0–10 cm and 10–20 cm. Notes: Subplots show the average soil NH_4_^+^-N, NO_3_^−^-N and TIN over the study period for each type of forest. Lowercase letters indicate significant differences among forest types (*p* < 0.05). The values are followed by mean ± SD. BF, *Betula platyphylla* forest; FF, *Fraxinus mandschurica* forest; PF, *Populus davidiana* forest.

Soil NH_4_^+^-N, NO_3_^−^-N, and TIN across different forests at both 0–10 cm and 10–20 cm soil layers consistently increased initially and then decreased during the study period. NH_4_^+^-N and TIN contents reached their highest levels in June, while that of NO_3_^−^-N was the highest in July (*p* < 0.05, Figure 1). NH_4_^+^-N, NO_3_^−^-N, and TIN contents showed significant differences between the two soil layers (Figure 1), with consistently higher values observed in the 0–10 cm soil layer than in the 10–20 cm soil layer (*p* < 0.05). The average contents of NH_4_^+^-N, NO_3_^−^-N, and TIN in the 0–10 cm soil layer all ranked in the order of FF > BF > PF (*p* < 0.05). In the 10–20 cm soil layer, PF had lower average NH_4_^+^-N, NO_3_^−^-N, and TIN contents than FF and BF, with no significant difference between FF and BF.

### 2.2. Soil N Mineralization Rate

**Figure 2:**
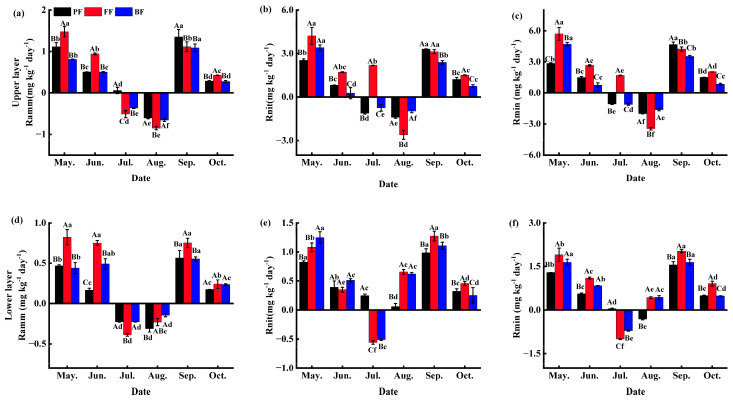
Seasonality dynamics of Ramm (**a**,**d**), Rnit (**b**,**e**), and Rmin (**c**,**f**) among the three types of forest at depths of 0–10 cm and 10–20 cm. Notes: Uppercase letters indicate significant differences between each forest type in each soil layer; lowercase letters indicate significant differences among the different months (*p* < 0.05). The values are followed by mean ± SD. BF, *Betula platyphylla* forest; FF, *Fraxinus mandschurica* forest; PF, *Populus davidiana* forest.

There were significant temporal variations in Ramm, Rnit, and Rmin in both soil layers among all forest types during the study period, with positive values in May, June, September, and October. At both soil layers, Ramm, Rnit, and Rmin showed significantly greater values in May and September than in the other sampling months (*p* < 0.05). The accumulation Rmin was highest in FF (334.05 ± 58.98 mg kg^−1^), followed by PF (201.51 ± 35.24 mg kg^−1^) and BF (147.10 ± 35.67 mg kg^−1^) (*p* < 0.05, Figure 2, Table 1). Similarly, the average soil net mineralization rate ranked as FF (2.17 ± 0.04 mg kg^−1^ d^−1^) > PF (1.31 ± 0.36 mg kg^−1^ d^−1^) > BF (0.95 ± 0.40 mg kg^−1^ d^−1^) (*p* < 0.05, Figure 2, Table 1).

### 2.3. Soil MBC, MBN and MBP

**Figure 3:**
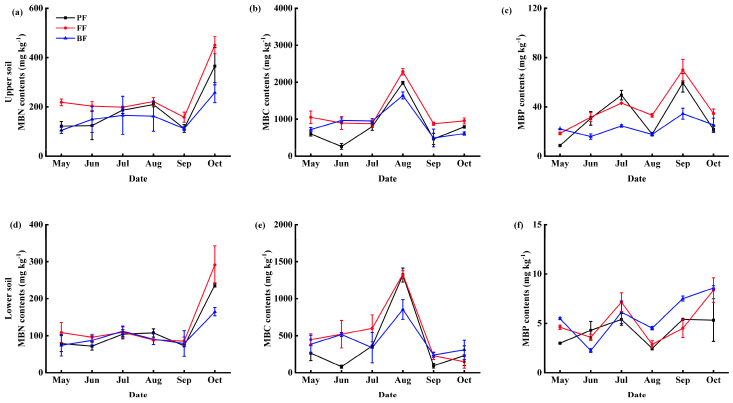
Seasonal dynamics of soil MBN, MBC and MBP contents at 0–10 cm and 10–20 cm depths under different forest types. Soil MBN content (**a**,**d**), MBC content (**b**,**e**), MBP content (**c**,**f**). BF, *Betula platyphylla* forest; FF, *Fraxinus mandschurica* forest; PF, *Populus davidiana* forest.

Soil MBN contents in PF, FF, and BF in both soil layers all showed fluctuating upward trends during the study period. Across all forest types, soil MBC contents showed a unimodal temporal pattern at both depths, reaching maximum values in August (*p* < 0.05) (Figure 3). MBP dynamics differed by soil depth across the three forests, showing a single September peak in the surface soil and a fluctuating rise in the deeper soil. Among the three forest types, soil MBN, MBC, and MBP contents showed significant vertical differences, with greater values in the 0–10 cm soil layer compared with the 10–20 cm soil layer (*p* < 0.05). The MBN, MBC, and MBP contents in the 0–10 cm soil layer were significantly higher in FF than in BF and PF (*p* < 0.05). In the 10–20 cm soil layer, these microbial biomass indices showed no significant differences between FF and BF, whereas their values were significantly higher in FF than in PF (*p* < 0.05) (Table 2).

### 2.4. Soil Microbial Community Composition

**Figure 4:**
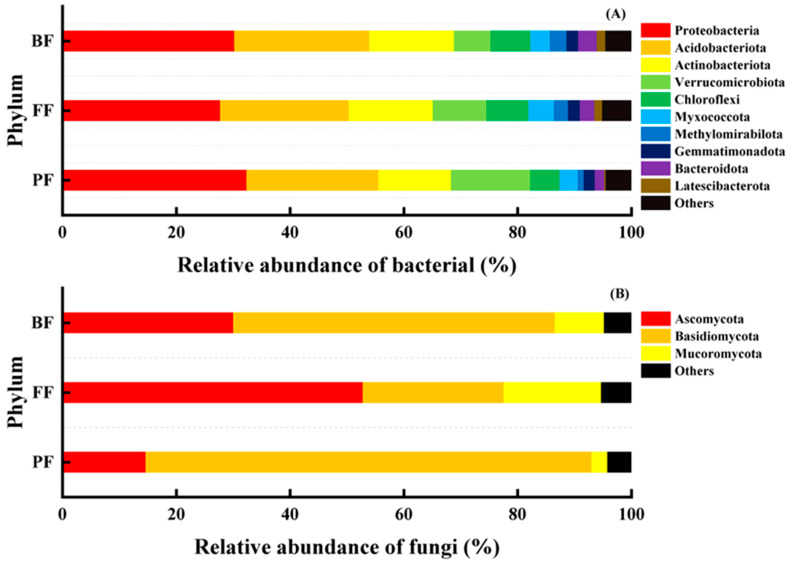
The relative abundances of soil bacteria communities (**A**) and fungal communities (**B**) at the phylum level. Groups with relative abundances greater than 1% are presented, while those with relative abundances lower than 1% are combined into the “other” group. BF, *Betula platyphylla* forest; FF, *Fraxinus mandschurica* forest; PF, *Populus davidiana* forest.

**Figure 5:**
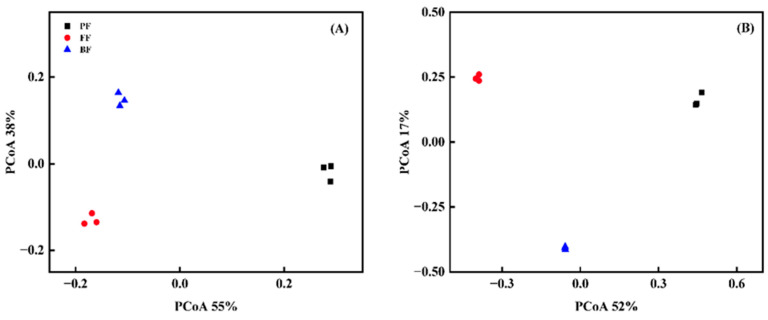
PCoA of soil bacterial (**A**) and fungal (**B**) beta diversities, based on the Bray–Curtis distances among different forest types. BF, *Betula platyphylla* forest; FF, *Fraxinus mandschurica* forest; PF, *Populus davidiana* forest.

The soil bacterial community among the three forest types was dominated by *Proteobacteria*, *Acidobacteriota*, *Actinobacteriota*, *Verrucomicrobiota*, and *Chloroflexi* (Figure 4A). Similarly, *Ascomycota*, *Basidiomycota*, and *Mucoromycota* represented the predominant fungal phyla, whereas other phyla showed relatively low abundances (Figure 4B). Bray–Curtis distance-based principal coordinate analysis (PCoA) revealed forest type-dependent ordination patterns in bacterial and fungal communities (Figure 5). Bacterial communities overlapped substantially between FF and BF, in contrast to the clear clustering pattern observed for fungal communities among forest types.

### 2.5. Rmin in Relation to Environmental Factors and Microbial Community

**Figure 6:**
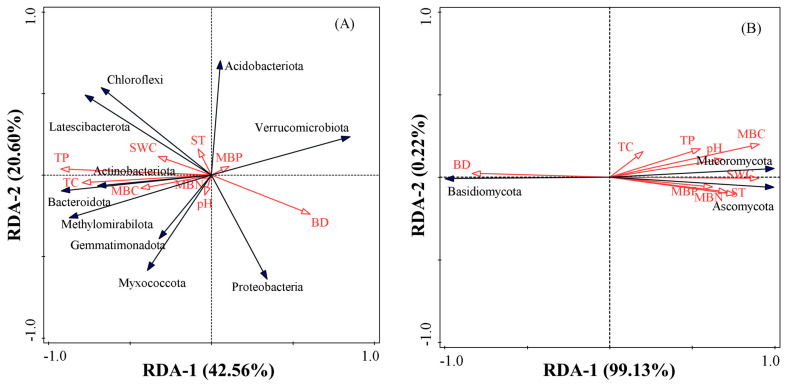
RDA of the abundant bacterial communities (**A**) and fungal communities (**B**) at the phylum level and soil properties for the samples from three different broad-leaved forests. Notes: ST, soil temperature; SWC, soil water content; MBC, microbial biomass carbon; MBN, microbial biomass nitrogen; MBP, microbial biomass phosphorus; BD, bulk density; TP, total phosphorus; TC, total carbon.

Redundancy analysis (RDA) identified key factors impacting the microbial community (Figure 6). The cumulative variance explained by the two main ordination axes was 63.16% for bacterial communities and 99.35% for fungal communities. Soil BD, SWC, ST, and TP explained large proportions of variation in bacterial community composition (*p* < 0.05, Table S1). For the fungal community, ST and SWC were the strongest explanatory factors, contributing 57.5% and 36.4% (*p* = 0.002), respectively. Other factors explained fewer variations in the soil microbial community (Table S2).

**Figure 7:**
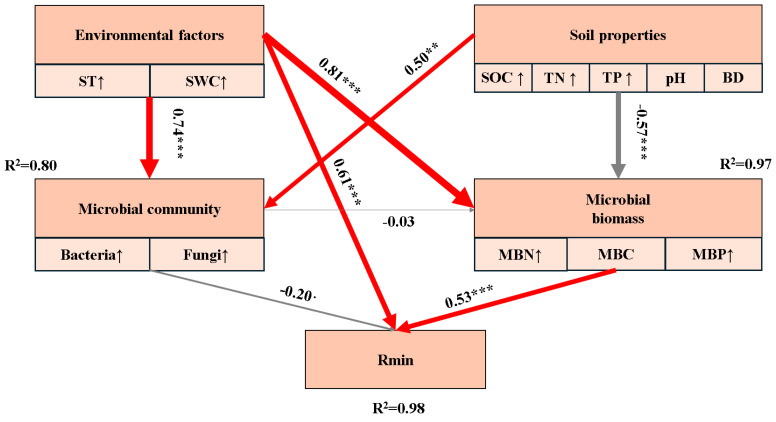
SEM depicting the influence of environmental factors and soil properties on microbial communities and Rmin. Notes: Results of model fitting: χ2 = 1.001, *p* = 0.996; df = 2; RMSEA = 0.000; CFI = 1.000; GFI = 1.000; AIC = 26.008. Red arrows indicate positive effects; gray arrows represent negative effects. Multiple-layer rectangles represent the first component from the PCoA conducted for the environmental factors, soil properties, microbial community and microbial biomass. The arrows “↑” indicate positive significant correlations between the variables and the first component from the PCoA. The environmental factors include ST and SWC; the soil properties include the TP, TN, SOC, BD and pH; the microbial community includes the bacteria and fungi; the microbial biomass includes the MBC, MBN and MBP. R^2^ values represent the proportion of variance explained for each variable. The values beside the arrow represent standardized coefficient. Significance of the correlation is indicated at the 0.001 (***), 0.01 (**) and 0.1 (·) level.

The results of SEM further summarized the relationships among the environmental factors, soil properties, microbial communities, microbial biomass, and Rmin (Figure 7). Overall, the fitted SEM yielded an R^2^ of 0.98 for Rmin among three forests. Environmental factors, microbial community, and microbial biomass directly affected Rmin. Environmental factors also were indirectly associated with Rmin through the positive association with microbial community and microbial biomass. However, soil properties showed only indirect associations with Rmin through the positive association with microbial community and the negative association with microbial biomass. Meanwhile, microbial community showed a marginally negative association with Rmin (significant at the *p* < 0.1 level), and microbial biomass showed a significant positive association with Rmin (*p* < 0.05).

## 3. Discussion

### 3.1. Effects of Forest Types on Rmin

Significantly higher soil Rmin was observed in FF compared with PF and BF (*p* < 0.05), although no significant difference was detected between PF and BF. Such differences in soil Rmin may be partly explained by variations in soil BD and SWC among the three forest types. Previous research has indicated that soil BD could regulate soil aeration by affecting soil porosity, and lower soil BD could improve soil aeration, which in turn affects microbial growth, leading to a higher Rmin [41]. The soil BD was significantly lower in FF than in PF and BF, and the relatively higher BD in PF and BF may be associated with their lower Rmin. Meanwhile, higher SWC could promote litter decomposition and release more SOC and available N (NH_4_^+^-N, NO_3_^−^-N) to the soil, which could supply sufficient substrates and nutrients for soil microbes and, in turn, increase Rmin [42]. Our study showed that the SWC in FF was significantly higher than that in PF and BF, which could also explain the higher Rmin in FF than in PF and BF. Consistent with previous findings, Rmin decreased significantly with soil depth, showing lower values in the 10–20 cm soil layer [43]. This difference may also be attributed to the more favorable environment, lower BD, and higher soil organic matter content in the 0–10 cm soil layer, which supports microbial growth, leading to a high Rmin. Interestingly, nitrification rate was the dominant process in our study (Table 1 and Figure 2). Previous studies have suggested that there are significant effects of ST on both Ramm and Rnit, and nitrifying microorganisms are more sensitive to high temperature than ammonifying microbes with ST exceeding 15 °C and SWC exceeding 30% [44,45]. In our study, the average ST across all forest types consistently exceeded 15 °C, and SWC remained above 30% during the study period, which explained the significantly higher Rnit than Ramm.

During the study period, Rmin in the three forest types exhibited V-shaped monthly dynamics. Higher Rmin in the initial and final sampling periods may be partly associated with seasonal shifts in plant N demand. It has been reported that plants generally take up less inorganic N during the early and late stages of the growing season [46]. Therefore, the reduced plant demand during these periods may have allowed a greater proportion of mineralized inorganic N to accumulate in the soil, thereby contributing to the higher Rmin values. In contrast, the negative Rmin values observed in July and August indicate that inorganic N consumption or loss exceeded net N production during this period. This pattern may be partly explained by enhanced microbial N demand under relatively high ST, which could promote the assimilation of NH_4_^+^-N and NO_3_^−^-N into microbial biomass when microbial immobilization exceeded gross mineralization [47]. In addition, the relatively high SWC in July and August (Figure S1) suggests that soil aeration within the incubation cores may have been partially restricted, potentially promoting localized low-oxygen microsites and denitrification, thereby reducing the extractable inorganic N pool [48].

### 3.2. Effects of Forest Types on Microbial Composition

In this study, the dominant bacterial phyla across the three forest types were *Proteobacteria*, *Acidobacteriota*, *Actinobacteriota*, *Verrucomicrobiota*, and *Chloroflexi*. For fungi, *Ascomycota*, *Basidiomycota*, and *Mucoromycota* (Figure 4 and Figure 5) were the predominant phyla. These findings are in agreement with previous reports [49]. These shared characteristics could provide soil bacteria with comparable carbon sources, nutrient conditions, and niche environments, thereby helping to maintain similar dominant bacterial groups [20]. In addition, bacterial phyla such as *Proteobacteria*, *Acidobacteriota*, and *Actinobacteriota* are widely distributed and commonly dominant in forest soils, indicating their strong adaptability to forest soil environments [49]. Therefore, similar soil habitat conditions may help explain why the three broadleaf forests shared the same dominant bacterial phyla and exhibited comparable abundance patterns. Although the three broad-leaved forests shared several dominant microbial phyla, the relative abundances of some dominant taxa still varied among forest types. This phenomenon may be attributed to several factors. On one hand, aboveground vegetation can affect soil microbial communities through root exudates and litter inputs [50]. Variations in litter quality and root traits can shape microbial diversity and community structure in soils [51]. On the other hand, the soil microenvironment could also affect the soil microbial community [52]. Forest type-related differences in SWC, ST, and pH may partly account for the observed shifts in soil microbial community structure and diversity.

FF had significantly higher MBN, MBC and MBP than both PF and BF (Figure 3 and Table 2). This pattern may be linked to soil pH differences among the three forests. Changes in pH can alter microbial community composition and metabolic activity, thereby affecting soil microbial biomass [53]. The augmentation of soil acidification frequently reduced soil microbial biomass [41]. The relatively higher soil pH in FF may provide more favorable conditions for microbial growth, which could help explain the greater microbial biomass in this forest type. Microbial biomass declined with increasing soil depth, probably because microbial activity strongly depends on the availability of soil substrates [52]. This vertical pattern may be further explained by the greater accumulation of litter-derived substrates in the surface soil, which could enhance microbial activity and ultimately increase microbial biomass [54]. Moreover, shifts in soil microenvironmental conditions with increasing soil depth may further constrain microbial growth and contribute to the lower microbial biomass in deeper soil layers [55]. Microbial biomass also showed clear monthly variations across the three forest types. The fluctuating increase in MBN and MBP and the unimodal pattern of MBC suggest that microbial C, N, and P pools may respond differently to seasonal environmental changes. These temporal patterns may be mainly associated with seasonal changes in SWC, ST, and substrate availability. Moreover, the effects of SWC and ST on microbial diversity and community composition may further contribute to the observed variation in microbial biomass [56,57].

### 3.3. Key Factor Regulating Rmin

Prior studies have suggested that ST and SWC are major environmental drivers of soil N mineralization [58]. In our study, environmental factors represented by ST and SWC were significantly correlated with Rmin and showed direct associations with Rmin (Figure 7, Table S3). Environmental factors and soil properties may be indirectly associated with Rmin through their associations with microbial communities and microbial biomass (Figure 7), and soil BD and pH showed significant relationships with Rmin (Table S3).

In this study, microbial community and microbial biomass could directly affect Rmin. This result partly agreed with our second hypothesis. Soil dominant bacterial phyla showed no significant differences in relative abundance among forest types (Tables S4 and S6), which led to no significant correlations between bacterial phylum abundances and Rmin. However, dominant fungal phyla relative abundance differed significantly among forest types (Table S5). Specifically, higher abundances of *Ascomycota* and *Mucoromycota* corresponded to increased Rmin (*p* < 0.05), whereas *Basidiomycota* was inversely related to Rmin (*p* < 0.05, Table S7). *Ascomycota* plays a pivotal role in soil Rmin via organic nitrogen decomposition [59]. Increase in soil *Ascomycota* abundance could stimulate organic N decomposition and the release of available N (NH_4_^+^-N), which directly drives the enhancement of soil Rmin. This may explain the relatively higher Rmin observed in FF in our study. *Mucoromycota* acts as a pioneer decomposer, rapidly degrading labile carbon compound. Thus, *Mucoromycota* efficiently decomposes organic N, thereby significantly accelerating Rmin [60]. Together with *Ascomycota*, the contribution of *Mucoromycota* may further support the higher Rmin observed in FF. In contrast, *Basidiomycota* taxa preferentially decompose recalcitrant soil organic carbon [29]. Furthermore, a higher relative abundance of *Basidiomycota* might inhibit the activities of *Ascomycota* and *Mucoromycota* via resource competition [61], ultimately leading to a reduction in soil Rmin. Therefore, the higher *Basidiomycota* abundance in PF and BF may partly explained their lower Rmin in our study. Moreover, differences in microbial biomass reflect how microorganisms utilize inorganic N, which could also lead to the significant difference in Rmin [62,63]. Microbial biomass regulates microbial basal respiration by affecting microbial activity, turnover rate and substrate utilization capacity, ultimately influencing Rmin [64,65,66].

## 4. Materials and Methods

### 4.1. Study Area

The study was conducted at Xiaoling, Heilongjiang, Northeast China, within the Zhangguangcai Mountain range (127° E, 45° N). The local climate belongs to the continental monsoon type. Mean annual temperature is approximately 2.0 °C, while yearly precipitation ranges between 550 and 700 mm, with about 85% occurring during the growing season. The annual frostless period lasts about 130 days, and the average altitude is 570 m above sea level [67]. Alfisols are the dominant soil type [68]. *Pinus koraiensis* Siebold et Zucc. forests represent the zonal vegetation in this area, and other forest types, including *Betula platyphylla* Suk., *Populus davidiana* Dode, *Larix gmelinii* (Rupr.) Kuzen., and *Fraxinus mandshurica* Rupr. stands, are also widely distributed.

### 4.2. Site Selection and Experimental Design

To evaluate how forest type regulates soil Rmin and identify the environmental and microbial mechanisms involved, we selected three typical broad-leaved forest types, specifically *P. davidiana* forest (PF), *F. mandschurica* forest (FF), and *B. platyphylla* forest (BF), with a distance exceeding 1 km. The sampling design included nine plots in total. For each forest type, three 20 m × 20 m plots were established within the area dominated by that forest type, and the plots were separated by at least 20 m to reduce spatial autocorrelation [69]. Within each plot, three soil sampling points were selected for subsequent soil collection. Table 3 and Table 4 summarize the main characteristics of the three broad-leaved forest types.

### 4.3. Soil Collection

From May to October 2024, soil Rmin in each plot was assessed on six occasions by applying the in situ undisturbed buried-core method. Three sampling positions were uniformly distributed along the right diagonal of each plot. During each sampling campaign, two PVC cylinders (5 cm diameter × 22 cm length) were installed vertically in the soil at each sampling position. One PVC tube was retrieved immediately and used for measuring the initial inorganic N concentration, while another PVC tube remained in the field for in situ incubation until the next sampling campaign, with an incubation period of approximately 30 d. The PVC tubes were placed at a soil depth of 20 cm, and their upper ends were sealed with permeable plastic film, which allowed air exchange but restricted water input and insect disturbance. Meanwhile, another three soil profiles were randomly set within each plot to collect soil samples at both 0–10 cm and 10–20 cm soil layers for soil physicochemical property analysis. Soil temperature (ST) was measured concurrently using a portable thermometer (Delta TRAK, Pleasanton, CA, USA) at each plot. Soil samples were transported to the laboratory and divided into two parts. One section was stored at 4 °C for the determination of soil NH_4_^+^-N, NO_3_^−^-N, and soil microbial biomass carbon (MBC), nitrogen (MBN), and phosphorus (MBP) [70]. Another section was air-dried at room temperature, and sieved to a size of 2 mm for the analysis of soil pH, organic carbon (SOC) and total nitrogen (TN) [71].

Meanwhile, a separate soil sampling was collected in mid-July 2024 for soil microbial analysis. Before sampling, surface litter was cleared, and soils were then taken from three random positions in each plot. The 0–10 cm and 10–20 cm soil layers were sampled separately. For each plot, soils collected from the same depth interval were combined to produce one representative composite sample for each layer, resulting in two composite samples corresponding to the 0–10 and 10–20 cm intervals. The composite samples were sealed in sterile sample tubes and transported to the laboratory under dry-ice cooling. The microbial community analysis was used to forest type differences rather than seasonal dynamics of Rmin.

### 4.4. Soil Chemical Analyses

KCl extracts were analyzed by flow injection analysis to quantify soil NH_4_^+^-N and NO_3_^—^N, which were extracted by 1 mol·L-1 KCl and measured by the AA3 AutoAnalyzer (Seal Analytical, Norderstedt, Germany). Soil total inorganic N (TIN) in soil was calculated from the combined concentrations of NH_4_^+^-N and NO_3_^−^-N. Soil water content (SWC) was calculated gravimetrically from the mass loss of fresh soil after a 24 h drying period in a 105 °C oven. For each incubation period, the net nitrification rate (Rnit), net ammonification rate (Ramm), and net nitrogen mineralization rate (Rmin) were calculated from changes in NO_3_^−^-N, NH_4_^+^-N, and TIN concentrations, respectively [72]. The calculation formulas were as follows:

$$\Delta t=t_{i+1}-t_{i}$$

$$Rnit=\frac{C_{inc}\left(NO_{3}^{-}-N\right)-C_{ini}\left(NO_{3}^{-}-N\right)}{\Delta t}$$

$$Ramm=\frac{C_{inc}\left(NH_{4}^{+}-N\right)-C_{ini}\left(NH_{4}^{+}-N\right)}{\Delta t}$$

Rmin=Ramm+Rnit

where *i* denotes a given incubation period; *t_i_* and *t_i_*_+1_ represent the initial and post-incubation sampling times, respectively; Δ*t* represents the actual incubation interval in days; and *C_ini_* and *C_inc_* represent the inorganic N concentrations in the initial and incubated soil samples, respectively. Rnit, Ramm, and Rmin represent the net nitrification rate, net ammonification rate, and net nitrogen mineralization rate, respectively. TIN represents the sum of NH_4_^+^-N and NO_3_^−^-N. All rates were expressed as mg kg^−1^ d^−1^ on a dry soil mass basis. For the 0–20 cm layer, Rnit, Ramm, and Rmin were calculated as the arithmetic means of the corresponding values from the 0–10 cm and 10–20 cm layers, which had the same thickness. The chloroform fumigation–extraction method was used to quantify MBC, MBN, and MBP.

### 4.5. Soil Microbial Community Properties Assays

Soil DNA extraction was performed with the PowerSoil^®^ DNA Isolation Kit following the manufacturer’s instructions (MoBio Inc., Carlsbad, CA, USA). Extracted DNA was checked for integrity through agarose gel-based analysis, and its concentration was recorded using NanoDrop 1000 equipment (Thermo Fisher Scientific, Waltham, MA, USA). Bacterial and fungal communities were characterized through amplification of marker regions, namely 16S rRNA V3–V4 for bacteria and ITS1 for fungi, with primer pairs 341F/806R and ITS1F/ITS2R, respectively [73].

The bacterial PCR protocol began with 5 min at 95 °C, followed by 27 cycles of 30 s at 94 °C, 30 s at 55 °C, and 45 s at 72 °C. Amplification was completed with a 10 min extension at 72 °C. For fungal DNA amplification, thermal cycling included initial denaturation at 95 °C for 2 min, followed by 30 cycles at 95 °C, 55 °C, and 72 °C for 30 s each, and a final extension at 72 °C for 5 min. PCR amplification was performed in triplicate for each sample, and PCR products from the same sample were pooled, purified, quantified, and sequenced using an Illumina paired-end sequencing platform [74].

Raw paired-end reads were demultiplexed according to barcode information and then quality-filtered. Low-quality bases were trimmed using a Q20 threshold with a 10 bp sliding window, and reads shorter than 50 bp after trimming were discarded. Paired-end reads were merged according to their overlap, with a minimum overlap length of 10 bp and a maximum mismatch ratio of 0.2 in the overlap region. Exact barcode matching was required, and up to two primer mismatches were allowed. Chimeric sequences were removed using Usearch with combined de novo and reference-based approaches against the gold database.

Sequence data were processed using an OTU-based pipeline involving Trimmomatic, FLASH, Usearch, QIIME, and in-house Perl scripts. Operational taxonomic units (OTUs) were clustered at 97% sequence similarity. Representative OTU sequences were taxonomically assigned using the uclust algorithm. Bacterial OTUs were assigned against the default SILVA database, Release 138.1, whereas fungal OTUs were assigned against the UNITE fungal database, Release 8.2. Taxonomic information was summarized from domain to species level where possible. To minimize the influence of uneven sequencing depth among samples, bacterial and fungal OTU tables were separately normalized to the minimum sequencing depth across the samples included in the present analysis before downstream analyses. Therefore, bacterial and fungal communities were analyzed separately rather than pooled.

Sequence data generated in this study can be accessed through the NCBI BioProject database under accession number PRJNA1055189.

### 4.6. Data Analysis

Soil properties, microbial community composition, and Rmin were compared among forest types using one-way analysis of variance (one-way ANOVA), followed by Duncan’s post hoc test [75]. The effects of forest type, month, soil depth, and their interactions on Rmin were analyzed using a linear mixed-effects model, with plot nested within forest type as a random effect. Pearson correlation was used to evaluate pairwise associations among the variables. Statistical procedures were completed in SPSS 21.0 for Windows (SPSS Institute Inc., Chicago, IL, USA), using *p* < 0.05 as the significance criterion. Origin 2024 software (OriginLab Corporation, Northampton, MA, USA) was applied for figure preparation. The relationships between microbial community composition and soil properties were assessed by redundancy analysis (RDA) using Canoco 4.5 (Microcomputer Power, Ithaca, NY, USA). Principal coordinate analysis (PCoA) was used to generate an ordination plot showing variation in soil microbial community composition across forest types.

### 4.7. Structural Equation Modeling

Structural equation modeling (SEM) was applied to evaluate the relationship among the environmental factors, soil properties, microbial communities, microbial biomass and Rmin. In our study, environmental factors were represented by the scores of the first dimension of the PCoA biplot based on ST and SWC. Soil properties were represented by the scores of the first dimension of the PCoA biplot based on soil BD, pH, TN, SOC, and TP. Microbial communities were represented by the scores of the first dimension of the PCoA biplot based on bacterial and fungal OTUs. Microbial biomass was represented by the scores of the first dimension of the PCoA biplot based on MBN, MBC, and MBP. The SEM was fitted in AMOS 26.0 (SPSS Inc., Chicago, IL, USA). Model adequacy required satisfaction of the chi-square and fit-index thresholds, namely *p* > 0.05 for the chi-square test, GFI > 0.90, CFI ≥ 0.95, and RMSEA < 0.05.

## 5. Conclusions

In this study, field in situ experiments were conducted in three broadleaf forests in Northeast China to clarify Rmin variations and their underlying regulatory mechanisms. Soil Rmin differed significantly among the three broadleaf forests, and nitrification was the primary pathway of soil N mineralization. SWC, ST, BD, and pH were identified as key regulators of soil Rmin (*p* < 0.05). The three forest types had similar microbial community composition but differed in microbial abundance and biomass. Variation in soil Rmin among three forests was directly influenced by environmental factors, microbial community and biomass, and indirectly affected by soil properties. This study revealed the regulatory pathways and mechanisms of forest types on soil Rmin. These findings deepened our understanding of soil nutrients in broadleaf forest ecosystems of Northeast China.

## Figures and Tables

**Table 1:** Soil net N mineralization accumulation and rate in different types of forest in the entire soil layer (0–20 cm) over the study period.

Forest Type	Rate			Accumulation		
	Ammonification	Nitrification	Mineralization	Ammonification	Nitrification	Mineralization
PF	0.34 ± 0.08 ab	0.96 ± 0.35 b	1.31 ± 0.36 b	53.09 ± 5.42 ab	148.42 ± 30.02 b	201.51 ± 35.24 b
FF	0.37 ± 0.05 a	1.80 ± 0.08 a	2.17 ± 0.04 a	56.98 ± 8.14 a	277.07 ± 50.67 a	334.05 ± 58.98 a
BF	0.29 ± 0.12 b	0.67 ± 0.38 b	0.95 ± 0.40 b	44.45 ± 5.94 b	102.65 ± 30.54 b	147.10 ± 35.67 b

Note: Lowercase letters indicate significant differences between forest types in each index (*p* < 0.05). The values are followed by mean ± SD. BF, *Betula platyphylla* forest; FF, *Fraxinus mandschurica* forest; PF, *Populus davidiana* forest.

**Table 2:** Soil MBN, MBC and MBP contents in both soil layers among different forests during the study period.

Forest Type	MBN Content (mg kg^−1^)		MBC Content (mg kg^−1^)		MBP Content (mg kg^−1^)	
	0–10 cm	10–20 cm	0–10 cm	10–20 cm	0–10 cm	10–20 cm
PF	195.4 ± 24.0 b	114.7 ± 5.1 b	833.9 ± 6.2 c	380.8 ± 16.2 b	31.1 ± 0.4 b	4.5 ± 0.1 b
FF	250.5 ± 12.0 a	129.8 ± 8.6 a	1156.2 ± 6.3 a	486.6 ± 39.3 a	38.4 ± 1.8 a	5.1 ± 0.4 a
BF	156.1 ± 4.7 c	116.5 ± 4.8 ab	885.4 ± 20.1 b	440.5 ± 68.9 ab	23.3 ± 0.6 c	5.7 ± 0.2 a

Note: Lowercase letters indicate significant differences between forest types in each index (*p* < 0.05). The values are followed by mean ± SD. BF, *Betula platyphylla* forest; FF, *Fraxinus mandschurica* forest; PF, *Populus davidiana* forest.

**Table 3:** Site basic characteristics of the three forest types.

Characteristics	PF	FF	BF
Altitude (m)	307 ± 3	305 ± 4	305 ± 5
Slope degree (°)	15–20	15–20	15–20
Stand Age	55	47	53
Canopy Density	0.85	0.80	0.75
DBH (cm)	12.78 ± 4.03	12.34 ± 3.2	15.30 ± 5.41
Tree Height (m)	9.60 ± 1.04	9.97 ± 0.84	10.58 ± 0.53
ST (°C)	15.5 ± 0.1 ab	15.7 ± 0.2 a	15.3 ± 0.1 b
SWC (%)	41.86 ± 0.52 b	57.39 ± 0.97 a	40.46 ± 0.15 b
BD (g cm^−3^)	0.97 ± 0.15 a	0.73 ± 0.13 b	0.91 ± 0.05 a
TP (g kg^−1^)	0.33 ± 0.01 c	0.40 ± 0.02 b	0.45 ± 0.02 a
SOC (g kg^−1^)	23.57 ± 2.23 c	29.35 ± 1.78 b	41.74 ± 3.61 a
TN (g kg^−1^)	1.98 ± 0.10 b	2.12 ± 0.32 b	2.86 ± 0.13 a
C:N	10.56 ± 0.91 b	13.84 ± 1.01 a	11.56 ± 1.77 ab
pH	6.12 ± 0.05 b	6.35 ± 0.11 a	6.01 ± 0.10 b

Note: Values were characterized using data from the 0–10 cm soil layer. DBH, diameter at breast height; ST, soil temperature; SWC, soil water content; BD, bulk density; TP, total phosphorus; SOC, soil organic carbon; TN, total nitrogen, C:N, carbon to nitrogen ratio. BF, *Betula platyphylla* forest; FF, *Fraxinus mandschurica* forest; PF, *Populus davidiana* forest. Lowercase letters indicate significant differences between forest types in each index (*p* < 0.05). The values are followed by mean ± SD.

**Table 4:** Understory vegetation composition of the three forest types.

Forest Types	Understory Vegetation Composition
PF	*Rhododendron simsii* Planch *Adenophora capillaris* subsp. Paniculate *Rubus crataegifolius* Bunge *Eriophorum scheuchzeri* Hoppe
FF	*Rhododendron simsii* Planch *Acer leptophyllum* Fang *Rhamnus davurica* Pall. *Eriophorum scheuchzeri* Hoppe
BF	*Rhododendron simsii* Planch *Corylus mandshurica* Maxim *Spiraea salicifolia* L. *Euonymus verrucosus* var. *pauciflorus*(Maxim.) Rege

Note: BF, *Betula platyphylla* forest; FF, *Fraxinus mandschurica* forest; PF, *Populus davidiana* forest.

## Data Availability

The sequencing data of soil bacteria and fungi have been deposited in the National Center for Biotechnology Information (NCBI) under accession number PRJNA1055189.

## Supplementary Materials

The following supporting information can be downloaded at: https://www.mdpi.com/article/10.3390/plants15142138/s1, Figure S1. Soil temperature among different forest types. Figure S2. Seasonal variation in soil water content in different soil layers among different forest types. Table S1. The correlation and eigenvalue results of RDA (bacterial community). Table S2. The correlation and eigenvalue results of RDA (fungal community). Table S3. Correlations between Rmin, environmental factors and soil properties. Table S4. Differences in relative abundance of bacterial OTUs among forest types. Table S5. Differences in relative abundance of fungal OTUs among forest types. Table S6. Correlation analysis among Rmin, abiotic factors and bacterial communities. Table S7. Correlation analysis among Rmin, abiotic factors and fungal communities. Table S8. Relative abundances of all fungal phyla across different forest types. Table S9. Effects of forest type, month, soil depth, and their interactions on soil nitrogen mineralization rate based on a linear mixed-effects model.
