# Supplementary material for: Variations in Soil Nitrogen Mineralization Are Associated with Fungal Communities Across Broad-Leaved Forests in Northeast China
Source: Plants (Basel). 2026 Jul 10;15(14):2138. doi: 10.3390/plants15142138 (PMC13416194; doi:10.3390/plants15142138)
Supplement: Supplementary file 1 [file plants-15-02138-s001.zip › plants-4403622-supplementary.pdf]

Supplementary File(s)

Variations in soil nitrogen mineralization are associated with fungal communities across broad-leaved forests in Northeast China

Xu Cao [1,2], Lei Guo [1,2], Ruihan Xiao [1,2,3,*], Kexin Tong [1,2], Tao Liu [1,2], Minghan Lang [4], Beixing Duan [1,2,3,*]

[1] School of Hydraulic and Electric Power, Heilongjiang University, Harbin 150080, China; coxi@s.hlju.edu.cn; guolei@s.hlju.edu.cn (L.G.); xiaoruihan@hlju.edu.cn (R.X.); 2022074@hlju.edu.cn (K.T.); 2002219@hlju.edu.cn (T.L.)
[2] International Joint Laboratory of Hydrology and Hydraulic Engineering in Cold Regions of Heilongjiang Province, Harbin 150080, China
[3] Post-doctoral Mobile Research Station of Ecology, Heilongjiang University, Harbin 150080, China
[4] Liaoning Zhanggutai Desert Ecosystem Research Station, Liaoning Institute of Sandy Land Control and Utilization, Fuxin 123000, China; 18804502009@163.com
* Correspondence: xiaoruihan@hlju.edu.cn, duanbx@hlju.edu.cn; Tel.: 86-182-4605-7664, 86-150-4610-5698

S1 Additional Materials

S1.1 Soil temperature

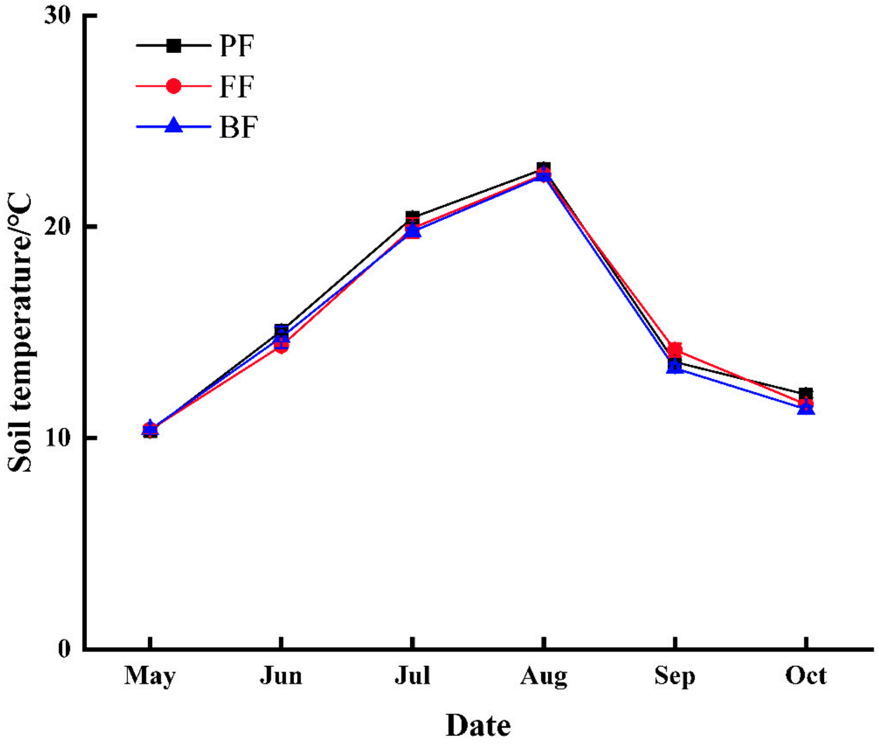

Figure S1. Soil temperature among different forest types.

S1.2 Soil water content

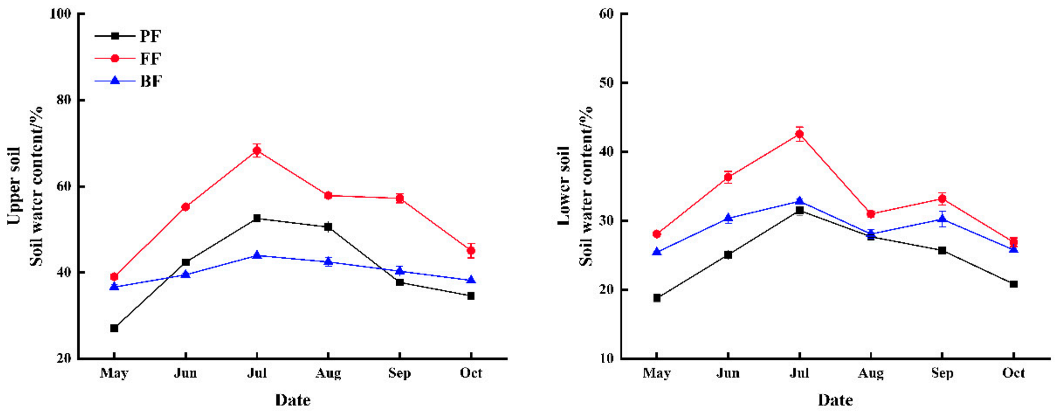

Figure S2. Seasonal variation in soil water content in different soil layers among different forest types.

S1.3 Eigenvalues and explained variance of RDA axes

Table S1. The correlation and eigenvalue results of RDA (bacterial community)

Name	Explains %	Contribution %	pseudo-F	P
BD	19.8	25.2	4.7	0.002
ST	15.1	19.3	4.2	0.008
SWC	16.8	21.5	5.9	0.002
pH	7.1	9.0	2.8	0.062
MBN	3.3	4.2	1.3	0.280
TC	4.1	5.2	1.7	0.186
TP	8.8	11.3	4.6	0.010
MBP	3.0	3.8	1.6	0.212
MBC	0.4	0.5	0.2	0.926

Table S2. The correlation and eigenvalue results of RDA (fungal community)

Name	Explains %	Contribution %	pseudo-F	P
ST	57.2	57.5	25.4	0.002
SWC	36.2	36.4	97.6	0.002
pH	1.5	1.5	5.0	0.036
TC	2.6	2.6	16.6	0.002
TP	0.9	0.9	8.0	0.008
MBN	0.2	0.2	1.9	0.178
BD	0.4	0.4	5.1	0.030
MBC	0.3	0.3	4.9	0.014
MBP	0.2	0.2	3.1	0.066

S1.4 Correlation analysis between environmental factors and Rmin.

Table S3. Correlations between Rmin, environmental factors and soil properties

	SWC	ST	TP	BD	SOC	TN	C:N	pH	Rmin
SWC	1	0.805**	0.097	-0.850**	-0.230	-0.279	0.743*	0.839**	0.972**
ST	0.805**	1	-0.220	-0.615	-0.410	-0.330	0.545	0.957**	0.858**
TP	0.097	-0.220	1	-0.150	0.880**	0.836**	0.370	-0.109	-0.134
BD	-0.850**	-0.615	-0.150	1	0.093	0.261	-0.445	-0.584	-0.821**
SOC	-0.230	-0.410	0.880**	0.093	1	0.926**	0.192	-0.329	-0.436
TN	-0.279	-0.330	0.836**	0.261	0.926**	1	0.163	-0.223	-0.474
C:N	0.743*	0.545	0.370	-0.445	0.192	0.163	1	0.689*	0.642
pH	0.839**	0.957**	-0.109	-0.584	-0.329	-0.223	0.689*	1	0.861**
Rmin	0.972**	0.858**	-0.134	-0.821**	-0.436	-0.474	0.642	0.861**	1

S1.5 Forest-type differences in bacterial and fungal OTUs

Table S4. Differences in relative abundance of bacterial OTUs among forest types

OTU ID	Acidobacteriota	Actinobacteriota	Bacteroidota	Chloroflexi	Gemmatimonadota	Latescibacterota	Methylomirabilota	Myxococcota	Proteobacteria	Verrucomicrobiota
PF	a	a	c	a	a	b	b	a	a	a
FF	a	a	b	a	a	a	a	a	a	ab
BF	a	a	a	a	a	a	a	a	a	b

Table S5. Differences in relative abundance of fungal OTUs among forest types

OTU ID	Ascomycota	Basidiomycota	Mucoromycota
PF	c	a	c
FF	a	c	a
BF	b	b	b

S1.6 Correlations of bacterial and fungal communities with all measured factors

Table S6. Correlation analysis among Rmin, abiotic factors and bacterial communities

	SWC	ST	BD	TP	SOC	MBC	pH	Rmin	MBP	MBN	Observed_species	Chao1	ACE	Shannon	Simpson	Pielou_J	Pd_faith	Acidobacteriota	Actinobacteriota	Bacteroidota	Chloroflexi	Gemmatimonadota	Latescibacterota	Methylomirabilota	Myxococcota	Proteobacteria	Verrucomicrobiota
SWC	1	.805**	-.850**	0.097	-0.23	0.615	.839**	.972**	.908**	.950**	0.612	0.581	0.555	0.512	0.466	0.472	0.566	-0.236	0.144	-0.023	0.391	0.081	0.258	0.18	0.572	-0.569	-0.063
ST	.805**	1	-0.615	-0.22	-0.41	0.346	.957**	.858**	.865**	.889**	0.229	0.186	0.192	0.14	0.084	0.108	0.227	-0.214	-0.384	-0.295	0.049	0.207	-0.135	-0.076	0.426	-0.314	0.244
BD	-.850**	-0.615	1	-0.15	0.093	-0.584	-0.584	-.821**	-.715*	-.815**	-0.637	-.734*	-.726*	-0.457	-0.337	-0.388	-0.522	0.393	-0.235	0	-0.192	0.292	-0.255	-0.219	-0.479	0.492	-0.081
TP	0.097	-0.22	-0.15	1	.880**	0.472	-0.109	-0.134	-0.293	-0.163	.795*	.681*	.680*	.881**	.865**	.897**	.833**	0.017	0.659	.942**	0.604	0.231	.750*	.870**	0.232	-0.318	-.739*
SOC	-0.23	-0.41	0.093	.880**	1	0.225	-0.329	-0.436	-0.594	-0.427	0.562	0.466	0.489	0.654	0.627	.674*	0.594	0.213	0.545	.823**	0.511	0.08	.699*	0.656	-0.12	-0.223	-0.579
MBC	0.615	0.346	-0.584	0.472	0.225	1	0.427	0.51	0.379	0.566	0.615	0.556	0.543	0.597	0.536	0.584	0.616	-0.067	0.275	0.24	0.51	-0.056	0.511	0.489	0.295	-.710*	-0.094
pH	.839**	.957**	-0.584	-0.109	-0.329	0.427	1	.861**	.839**	.880**	0.299	0.205	0.191	0.246	0.214	0.226	0.317	-0.262	-0.19	-0.186	0.134	0.321	-0.091	0.034	0.533	-0.31	0.078
Rmin	.972**	.858**	-.821**	-0.134	-0.436	0.51	.861**	1	.975**	.986**	0.419	0.416	0.392	0.299	0.252	0.255	0.363	-0.245	-0.026	-0.246	0.234	0.01	0.072	-0.024	0.511	-0.492	0.13
MBP	.908**	.865**	-.715*	-0.293	-0.594	0.379	.839**	.975**	1	.954**	0.249	0.247	0.223	0.137	0.107	0.099	0.198	-0.192	-0.189	-0.38	0.137	0.019	-0.063	-0.176	0.443	-0.413	0.234
MBN	.950**	.889**	-.815**	-0.163	-0.427	0.566	.880**	.986**	.954**	1	0.38	0.381	0.365	0.254	0.193	0.209	0.328	-0.221	-0.106	-0.301	0.211	-0.025	0.057	-0.047	0.453	-0.526	0.22
Observed_species	0.612	0.229	-0.637	.795*	0.562	0.615	0.299	0.419	0.249	0.38	1	.953**	.944**	.969**	.921**	.944**	.981**	-0.146	0.638	.721*	.671*	0.172	.765*	.763*	0.495	-0.534	-0.631
Chao1	0.581	0.186	-.734*	.681*	0.466	0.556	0.205	0.416	0.247	0.381	.953**	1	.996**	.859**	.783*	.813**	.895**	-0.279	0.624	0.622	0.501	0.003	.674*	.706*	0.502	-0.45	-0.512
ACE	0.555	0.192	-.726*	.680*	0.489	0.543	0.191	0.392	0.223	0.365	.944**	.996**	1	.846**	.762*	.799**	.886**	-0.248	0.573	0.617	0.495	-0.017	.682*	.695*	0.456	-0.46	-0.478
Shannon	0.512	0.14	-0.457	.881**	0.654	0.597	0.246	0.299	0.137	0.254	.969**	.859**	.846**	1	.983**	.996**	.987**	-0.033	0.652	.816**	.751*	0.3	.806**	.809**	0.443	-0.519	-.737*
Simpson	0.466	0.084	-0.337	.865**	0.627	0.536	0.214	0.252	0.107	0.193	.921**	.783*	.762*	.983**	1	.991**	.960**	0.011	.671*	.832**	.779*	0.406	.793*	.796*	0.448	-0.471	-.817**
Pielou_J	0.472	0.108	-0.388	.897**	.674*	0.584	0.226	0.255	0.099	0.209	.944**	.813**	.799**	.996**	.991**	1	.975**	0.005	0.648	.836**	.768*	0.342	.808**	.813**	0.421	-0.507	-.764*
Pd_faith	0.566	0.227	-0.522	.833**	0.594	0.616	0.317	0.363	0.198	0.328	.981**	.895**	.886**	.987**	.960**	.975**	1	-0.132	0.607	.784*	.685*	0.337	.749*	.829**	0.528	-0.486	-.714*
Acidobacteriota	-0.236	-0.214	0.393	0.017	0.213	-0.067	-0.262	-0.245	-0.192	-0.221	-0.146	-0.279	-0.248	-0.033	0.011	0.005	-0.132	1	-0.243	-0.106	0.58	-0.215	0.476	-0.391	-.817**	-0.533	0.164
Actinobacteriota	0.144	-0.384	-0.235	0.659	0.545	0.275	-0.19	-0.026	-0.189	-0.106	0.638	0.624	0.573	0.652	.671*	0.648	0.607	-0.243	1	0.662	0.411	0.038	0.527	0.609	0.376	-0.106	-.721*
Bacteroidota	-0.023	-0.295	0	.942**	.823**	0.24	-0.186	-0.246	-0.38	-0.301	.721*	0.622	0.617	.816**	.832**	.836**	.784*	-0.106	0.662	1	0.461	0.438	0.608	.906**	0.35	-0.049	-.884**
Chloroflexi	0.391	0.049	-0.192	0.604	0.511	0.51	0.134	0.234	0.137	0.211	.671*	0.501	0.495	.751*	.779*	.768*	.685*	0.58	0.411	0.461	1	0.087	.923**	0.319	-0.101	-.818**	-0.45
Gemmatimonadota	0.081	0.207	0.292	0.231	0.08	-0.056	0.321	0.01	0.019	-0.025	0.172	0.003	-0.017	0.3	0.406	0.342	0.337	-0.215	0.038	0.438	0.087	1	-0.076	0.466	0.558	0.31	-0.653
Latescibacterota	0.258	-0.135	-0.255	.750*	.699*	0.511	-0.091	0.072	-0.063	0.057	.765*	.674*	.682*	.806**	.793*	.808**	.749*	0.476	0.527	0.608	.923**	-0.076	1	0.47	-0.127	-.761*	-0.476
Methylomirabilota	0.18	-0.076	-0.219	.870**	0.656	0.489	0.034	-0.024	-0.176	-0.047	.763*	.706*	.695*	.809**	.796*	.813**	.829**	-0.391	0.609	.906**	0.319	0.466	0.47	1	0.6	-0.053	-.785*
Myxococcota	0.572	0.426	-0.479	0.232	-0.12	0.295	0.533	0.511	0.443	0.453	0.495	0.502	0.456	0.443	0.448	0.421	0.528	-.817**	0.376	0.35	-0.101	0.558	-0.127	0.6	1	0.214	-0.509
Proteobacteria	-0.569	-0.314	0.492	-0.318	-0.223	-.710*	-0.31	-0.492	-0.413	-0.526	-0.534	-0.45	-0.46	-0.519	-0.471	-0.507	-0.486	-0.533	-0.106	-0.049	-.818**	0.31	-.761*	-0.053	0.214	1	-0.074
Verrucomicrobiota	-0.063	0.244	-0.081	-.739*	-0.579	-0.094	0.078	0.13	0.234	0.22	-0.631	-0.512	-0.478	-.737*	-.817**	-.764*	-.714*	0.164	-.721*	-.884**	-0.45	-0.653	-0.476	-.785*	-0.509	-0.074	1

Table S7. Correlation analysis among Rmin, abiotic factors and fungal communities

	SWC	ST	BD	TP	SOC	MBC	pH	Rmin	MBP	MBN	Ascomycota	Basidiomycota	Mucoromycota	Observed_species	Chao1	ACE	Shannon	Simpson	Pielou_J	Pd_faith
SWC	1	.805**	-.850**	0.097	-0.23	0.615	.839**	.972**	.908**	.950**	.885**	-.883**	.878**	0.069	0.381	0.415	0.271	0.182	0.261	0.206
ST	.805**	1	-0.615	-0.22	-0.41	0.346	.957**	.858**	.865**	.889**	0.594	-0.58	0.534	-0.138	0.001	0.055	-0.119	-0.221	-0.135	-0.148
BD	-.850**	-0.615	1	-0.15	0.093	-0.584	-0.584	-.821**	-.715*	-.815**	-.789*	.827**	-.812**	-0.27	-0.378	-0.404	-0.345	-0.311	-0.345	-0.279
TP	0.097	-0.22	-0.15	1	.880**	0.472	-0.109	-0.134	-0.293	-0.163	0.516	-0.532	0.548	.938**	.817**	.760*	.954**	.947**	.945**	.903**
SOC	-0.23	-0.41	0.093	.880**	1	0.225	-0.329	-0.436	-0.594	-0.427	0.242	-0.245	0.23	.855**	0.661	0.592	.843**	.870**	.842**	.796*
MBC	0.615	0.346	-0.584	0.472	0.225	1	0.427	0.51	0.379	0.566	.672*	-.725*	.721*	0.52	0.359	0.272	0.562	0.521	0.586	0.461
pH	.839**	.957**	-0.584	-0.109	-0.329	0.427	1	.861**	.839**	.880**	.667*	-0.644	0.612	-0.112	0.042	0.093	-0.004	-0.118	-0.01	-0.123
Rmin	.972**	.858**	-.821**	-0.134	-0.436	0.51	.861**	1	.975**	.986**	.757*	-.756*	.748*	-0.107	0.183	0.232	0.046	-0.04	0.039	-0.005
MBP	.908**	.865**	-.715*	-0.293	-0.594	0.379	.839**	.975**	1	.954**	0.621	-0.619	0.616	-0.25	0.058	0.122	-0.138	-0.225	-0.149	-0.143
MBN	.950**	.889**	-.815**	-0.163	-0.427	0.566	.880**	.986**	.954**	1	.728*	-.732*	.710*	-0.082	0.114	0.146	0.02	-0.065	0.018	-0.037
Ascomycota	.885**	0.594	-.789*	0.516	0.242	.672*	.667*	.757*	0.621	.728*	1	-.992**	.982**	0.464	.722*	.729*	.669*	0.592	0.655	0.593
Basidiomycota	-.883**	-0.58	.827**	-0.532	-0.245	-.725*	-0.644	-.756*	-0.619	-.732*	-.992**	1	-.993**	-0.512	-.711*	-.714*	-.684*	-0.613	-.673*	-0.606
Mucoromycota	.878**	0.534	-.812**	0.548	0.23	.721*	0.612	.748*	0.616	.710*	.982**	-.993**	1	0.513	.722*	.730*	.692*	0.622	.682*	0.611
Observed_species	0.069	-0.138	-0.27	.938**	.855**	0.52	-0.112	-0.107	-0.25	-0.082	0.464	-0.512	0.513	1	.823*	.768*	.942**	.935**	.936**	.906**
Chao1	0.381	0.001	-0.378	.817**	0.661	0.359	0.042	0.183	0.058	0.114	.722*	-.711*	.722*	.823*	1	.986**	.877**	.856**	.845**	.942**
ACE	0.415	0.055	-0.404	.760*	0.592	0.272	0.093	0.232	0.122	0.146	.729*	-.714*	.730*	.768*	.986**	1	.826**	.799**	.790*	.885**

Shannon	0.271	-0.119	-0.345	.954**	.843**	0.562	-0.004	0.046	-0.138	0.02	.669*	-.684*	.692*	.942**	.877**	.826**	1	.992**	.997**	.923**
Simpson	0.182	-0.221	-0.311	.947**	.870**	0.521	-0.118	-0.04	-0.225	-0.065	0.592	-0.613	0.622	.935**	.856**	.799**	.992**	1	.992**	.925**
Pielou_J	0.261	-0.135	-0.345	.945**	.842**	0.586	-0.01	0.039	-0.149	0.018	0.655	-.673*	.682*	.936**	.845**	.790*	.997**	.992**	1	.901**
Pd_faith	0.206	-0.148	-0.279	.903**	.796*	0.461	-0.123	-0.005	-0.143	-0.037	0.593	-0.606	0.611	.906**	.942**	.885**	.923**	.925**	.901**	1

S1.7 Relative abundance of fungi

Table S8. Relative abundances of all fungal phyla across different forest types

OUT ID	PF	FF	BF
Ascomycota	0.145845	0.527576	0.300002
Basidiomycota	0.783917	0.247856	0.564992
Chytridiomycota	0.000163	0.001793	0.002394
Eukaryota norank	0	0	0.0000713
Mucoromycota	0.027739	0.171057	0.08667
Olpidiomycota	0	0.00001018	0
Unclassified	0.041837	0.046758	0.045148
Zoopagomycota	0.000499	0.004951	0.000723

S1.8 Interaction effects on Rmin

Table S9. Effects of forest type, month, soil depth, and their interactions on soil nitrogen mineralization rate based on a linear mixed-effects model.

Source of variation	Sum of squares	Mean square	Num. df	Den. df	F value	P value	FDR-adjusted P value	Partial η^2
Forest type	7.40	3.70	2	72	228.93	< 0.001	< 0.001	0.86
Month	261.16	52.23	5	72	3229.61	< 0.001	< 0.001	0.99
Soil depth	22.68	22.68	1	72	1402.86	< 0.001	< 0.001	0.95
Forest type × Month	18.76	1.87	10	72	116.01	< 0.001	< 0.001	0.94
Forest type × Soil depth	3.42	1.71	2	72	105.96	< 0.001	< 0.001	0.74
Month × Soil depth	84.62	16.92	5	72	1046.50	< 0.001	< 0.001	0.98
Forest type × Month × Soil depth	21.63	2.16	10	72	133.75	< 0.001	< 0.001	0.94
